# Protein kinase C mediates hypoxia-induced long-term potentiation of NMDA neurotransmission in the visual retinocollicular pathway

**DOI:** 10.3389/fncel.2023.1141689

**Published:** 2023-02-24

**Authors:** Hanna Dumanska, Nikolai Veselovsky

**Affiliations:** Department of Neuronal Network Physiology, Bogomoletz Institute of Physiology, National Academy of Science of Ukraine, Kyiv, Ukraine

**Keywords:** retinocollicular pathway, hypoxia, long-term potentiation, protein kinase C, reversal of LTP, NMDA postsynaptic currents, decay time

## Abstract

The identification of processes and mechanisms underlying the early stage of hypoxic injury of the retinocollicular pathway may be beneficial for the future prevention and treatment of navigation, orientation, and visual attention impairments. Previously, we have demonstrated that short-term hypoxia led to long-term potentiation (LTP) of NMDA neurotransmission in the background of long-term depression of GABA_A_ retinocollicular transmission. Here, we sought to obtain insight into the mechanisms of hypoxia-induced LTP of NMDA retinocollicular neurotransmission and the role of the protein kinase C (PKC) signaling pathway in it. To investigate these, we recorded pharmacologically isolated NMDA transmission in cocultivated pairs of rat retinal ganglion cells and superficial superior colliculus neurons under normoxic and hypoxic conditions, using the paired patch-clamp technique and method of fast local superfusion. We tested the involvement of the PKC by adding the potent and selective inhibitor chelerythrine chloride (ChC, 5 μM). We observed that hypoxia-induced LTP of NMDA neurotransmission is associated with the shortening of current kinetics. We also found that the PKC signaling pathway mediates hypoxia-induced LTP and associated shortening of NMDA currents. The ChC completely blocked the induction of LTP by hypoxia and associated kinetic changes. Contrary effects of ChC were observed with already induced LTP. ChC led to the reversal of LTP to the initial synaptic strength but the current kinetics remain irreversibly shortened. Our results show that ChC is a promising agent for the prevention and treatment of hypoxic injuries of NMDA retinocollicular neurotransmission and provide necessary electrophysiological basics for further research.

## 1. Introduction

Pathogenesis of numerous diseases and traumas as well as several physiological states are associated with hypoxia (Biddlestone et al., 2015; Luo et al., 2022). The retinocollicular pathway, as part of the visual system, is extremely sensitive to oxygen deprivation (Wong-Riley, 2010). Lesions of this pathway lead to navigation, orientation, and visual attention deficits, and also could be involved in several neurological and psychiatric disorders such as attention deficit hyperactivity disorder, and autism (Brace et al., 2015; Mathis et al., 2015; Jure, 2022). In our research, we focus on the very early hypoxia-induced processes and their mechanisms as potential therapeutic targets serving to prevent lesions of the retinocollicular visual transmission.

We have shown previously, that hypoxia induces a significant shift in excitatory-inhibitory balance toward excitation. Oxygen deprivation led to long-term potentiation (LTP) of NMDA transmission and persistent increase in the amplitude and occurrence frequency of spontaneous NMDA events in the background of long-term depression of GABA_A_ retinocollicular transmission (Dumanska and Veselovsky, 2019). Such pathologically-induced functional alterations may reflect structural changes in NMDAR subunit composition. These receptors are extremely important in the development and refinement of the neurotransmission (Cull-Candy and Leszkiewicz, 2004; Rebola et al., 2010). The synaptic retinocollicular NMDARs contain NR2A and NR2B subunits at different developmental stages that determine the receptors distinct properties and functions (Townsend et al., 2004). The structural and functional alterations of NMDARs were observed in various pathological states and may contribute to molecular processes affecting cell survival or death (Lau and Zukin, 2007; Dewachter et al., 2009; Georgiou et al., 2010). Multiple intra and extracellular messengers, and enzymes regulate such alterations (Yaka et al., 2002; Lin et al., 2006). Among all, testing the involvement of the protein kinase C (PKC) signaling pathway in hypoxia-induced LTP of NMDA transmission seems to be the most promising prospect. Previous studies have shown that the PKC signaling pathway is involved in cellular response to hypoxia as well as in structural and functional alterations of NMDAR (Goldberg et al., 1997; Yan et al., 2000; Chen and Roche, 2007; Lee et al., 2007; Rebola et al., 2010; Kim et al., 2016). In this study, we tested the hypothesis that the protein kinase C (PKC) pathway might be involved in hypoxia-induced LTP NMDA retinocollicular transmission.

## 2. Materials and methods

In our experiments, we used an *in vitro* model of the visual retinocollicular pathway – primary coculture of rat retinal cells and superficial superior colliculus (SSC) neurons.

All manipulations with animals were performed in aseptic conditions in accordance with animal research regulations approved by the Ukrainian Academy of Science (in accordance with the European Convention for the Protection of Vertebrate Animals used for Experimental and other Scientific Purposes - Explanatory Report, 1986; World Medical Association Declaration of Helsinki, 1996; Convention for the Protection of Human Rights and Dignity of the Human Being with regard to the Application of Biology and Medicine: Convention on Human Rights and Biomedicine, 1997).

### 2.1. Coculture

The coculture was prepared as we previously described (Dumanska and Veselovsky, 2019). Briefly, the retinal and SSC tissues were obtained from pups P0-P1 of Wistar rats, both sexes. For this research we used 9 pups. After enzymatic and mechanical dissociation of the primary tissues, two suspensions of cells were placed in separate compartments of the originally-designed chamber for cocultivation in a Petri dish. The chamber consists of a silicon ring with a vertical glass baffle placed on the coverslip. One hour of incubation in a humidified atmosphere of 5 ± 0.5% CO_2_ at 37 ± 0.5 C was enough for cell adhesion to the coverslip. After that, the silicon ring was removed, and the cells were stored in the incubator for further cocultivation.

### 2.2. Electrophysiological recordings

In the coculture, we identified synaptically connected pairs of retinal ganglion cells (RGCs) and SSC neurons by their spatial location, morphological and electrophysiological characteristics (Moriton et al., 2013; Villalobos et al., 2018). The recordings were performed from synaptically connected pairs of RGCs and SSC at room temperature (20–24°C) using the paired whole-cell patch clamp technique. Pharmacologically isolated NMDA-mediated postsynaptic currents (PSCs) were evoked in SSC neurons by generation action potentials in presynaptic RGCs. Spontaneous currents were recorded in SSC neurons in the absence of presynaptic stimulation.

In all experiments, the extracellular solution contained (in mM): NaCl 140; KCl 3; CaCl_2_ 3; Hepes 20, and glucose 15 (Sigma-Aldrich); pH 7.4. For pharmacological isolation of NMDA currents, we added to the external solution dinitroquinoxaline-2,3(1H, 4H)-dione (DNQX, 20 μM) and bicuculline methiodide (10 μM). The internal pipette solution contained (in mM): potassium gluconate 155; EGTA 0.5; MgCl_2_ 1 and Hepes 20 (Sigma-Aldrich); pH 7.4. We tested the involvement of the PKC by adding 5 μM of chelerythrine chloride (ChC) to the external solution. Patch pipettes were prepared from borosilicate glass capillaries (World Precision Instruments, USA) with internal tip diameters 1.0–1.5 μm.

During the electrophysiological recordings, we constantly evaluated the quality of voltage clamping by monitoring the variations of the leakage current amplitude (I_leak_) and the time constant of the capacitive current (τ_cap_) recorded upon applications of short (10 ms) small-amplitude hyperpolarizing rectangular stimuli (– 10 mV). The data obtained were analyzed if variations of the τ_cap_ and I_leak_ values did not exceed 20 % of the mean value.

For both types of neurons, membrane potential varied from −50 to – 70 mV. Short-term hypoxic states did not lead to statistically significant changes in membrane potential (depolarization or hyperpolarization).

Data were recorded and digitized (10 kHz) using two Axopatch-1D amplifiers (CV-4 headstages, gain: × 1/100; 5 kHz cutoff low-pass 4-pole Bessel filter), Digidata 1322A and Clampex 9.0 software (Axon Instruments).

### 2.3. Modulation of short hypoxic states *in vitro*

Using the method of fast local superfusion (Veselovsky et al., 1996) we applied hypoxic solutions on synaptically -connected pairs of neurons during the electrophysiological recordings to mimic short-term hypoxic states *in vitro*. This method allowed us to control the area and speed of the application. The hypoxic solutions were obtained by saturation of the external solutions with nitrogen for 20 min just before the electrophysiological recordings. We used the next protocol for the experiments: first, we applied a normoxic external solution for 5 min – this period we called a control, then we switched to the hypoxic solution for 5 min – hypoxia, and then back to the normoxic external solution – reoxygenation.

### 2.4. Statistical analysis

Statistical analysis of data obtained has been done in Origin 8.5 Pro (OriginLab Corporation, USA) and Clampfit 9.0 (Axon Instruments, USA). The data is presented as mean ± SD. The decay time constants of the currents were fitted by a standard single-exponential function. We checked the normality of data sets using the Shapiro-Wilks test, the differences between two sets of values using two-sample *t*-test and the differences between the two functions using Kolmogorov-Smirnov criteria. The results of the *t*-test are represented as *t*-values (t), degrees of freedom (df), and *p*-values (p).

## 3. Results

**Figure 1:**
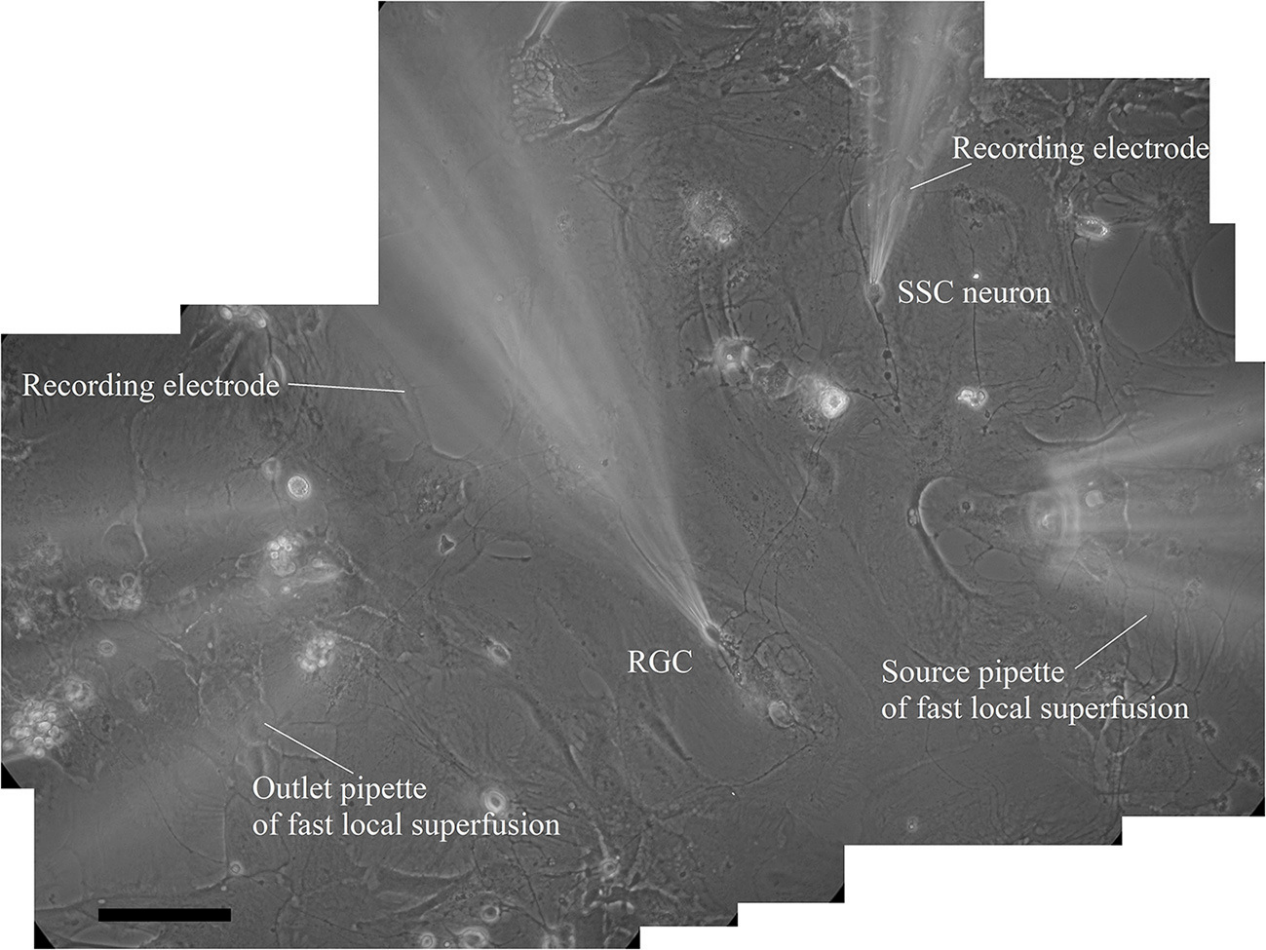
Reconstructed microphotography of synaptically connected couple of presynaptic retinal ganglion cell and postsynaptic superficial superior colliculus neuron in coculture during paired patch-clamp recording and fast local superfusion application on the 21st day *in vitro*; the scale marker corresponds to 100 μm.

In the coculture, each identified synaptically connected pair of RGCs and SSC neurons reflects a single fiber of the retinocollicular pathway (Figure 1). We examined 38 pairs of RGCs-SSC neurons. The evoked and spontaneous NMDA currents were identified by their kinetic and pharmacological characteristics (Furman and Crair, 2012).

**Figure 2:**
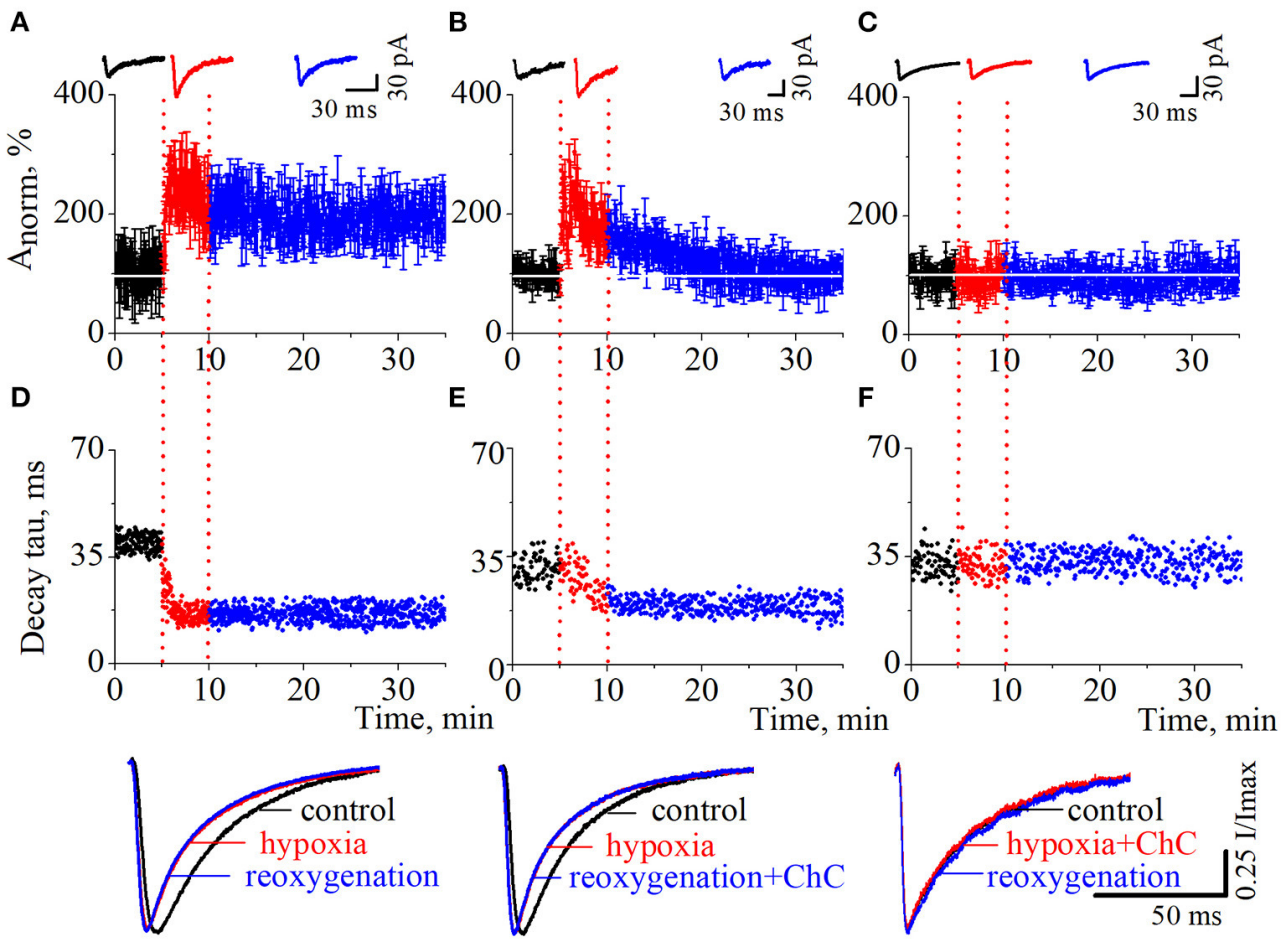
The effects of chelerythrine chloride (ChC) on hypoxia-induced LTP of NMDA synaptic neurotransmission. **(A–C)** The dynamics of the evoked postsynaptic current (PSCs) normalized average amplitudes with 5 min duration of the hypoxia application. The ChC (5 μM) was added during reoxygenation **(B)** and during hypoxia **(C)**. Representative recordings of the evoked PSCs are plotted against the corresponding period (control - black, hypoxia - red, and reoxygenation - blue) **(D–F)**. The dynamics of the currents decay time constants from **(A–C)**, respectively. Representative normalized currents are plotted bellow.

As we have reported before (Dumanska and Veselovsky, 2019), the application of a hypoxic solution for 5 min led to the long-term potentiation (LTP) of NMDA neurotransmission (Figure 2A, *n* = 8, unpublished data). PSCs displayed single exponential deactivation time course. We observed that hypoxia-induced LTP is associated with a rapid, irreversible and statistically extremely significant shortening of evoked PSCs – the decrease in current decay time constants (Figure 2D; control 39.7 ± 2.6 ms; hypoxia 16.7 ± 2.0 ms; reoxygenation 16.4 ± 2.5 ms; *t* = 85.6, *df* = 296, *p* < 0.0001 – hypoxia compare to control; *t* = 103.2, *df* = 897, *p* < 0.0001 – reoxygenation compare to control).

To investigate the role of the PKC signaling pathway in hypoxia-induced LTP we carried out two series of experiments. In the first, we added ChC (5 μM) to the external solution during the reoxygenation, and in the second – during hypoxia. In the first case, the hypoxia application successfully induced potentiation of the evoked PSCs but the presence of ChC during reoxygenation led to the decrease of the elevated amplitudes to the basal pre-LTP level (Figure 2B, *n* = 8). The dynamic of the PSCs decay time constants represents the irreversible and statistically extremely significant decrease during hypoxia and reoxygenation (Figure 2E; control 31.1 ± 4 ms; hypoxia 19.5 ± 3.2 ms; reoxygenation in the presence of ChC 19.2 ± 2.5 ms; *t* = 27.6, *df* = 296, *p* < 0.0001 – hypoxia compare to control; *t* = 46.8, *df* = 897, *p* < 0.0001 – reoxygenation compare to control). In the second case, the presence of ChC in the hypoxic solution completely blocked the hypoxia-induced LTP of NMDA transmission (Figure 2C) and associated changes in PSCs kinetics (Figure 2F; control 32.4 ± 3.9 ms; hypoxia in the presence of ChC 32.5 ± 3.9 ms; reoxygenation 32.4 ± 3.6 ms; *t* = 0.2, *df* = 296, *p* = 0.8 – hypoxia compare to control; *t* = 0, *df* = 897, *p* = 1 – reoxygenation compare to control.

**Figure 3:**
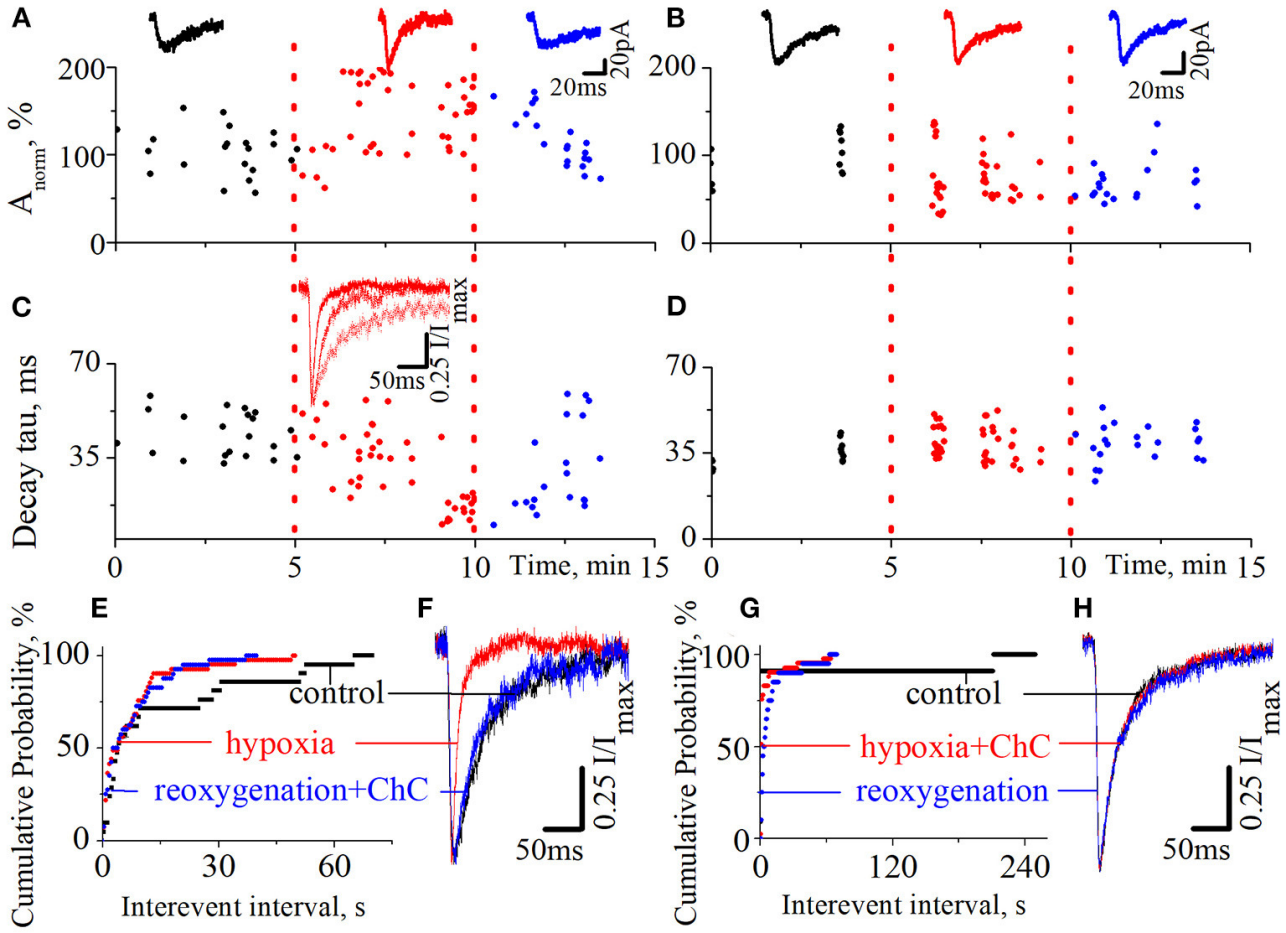
The effects of chelerythrine chloride (ChC) on the hypoxia-induced increase of spontaneous NMDA synaptic activity. **(A, B)** The dynamics of spontaneous currents normalized amplitudes with 5 min duration of hypoxia application. The ChC (5 μM) was added during reoxygenation **(A)** and during hypoxia **(B)**. The representative recordings of spontaneous currents are plotted against the corresponding period (control - black, hypoxia - red, and reoxygenation - blue) **(C, D)** The dynamics of currents decay time constants from **(A, B)**, respectively **(E, G)** The cumulative probability plots of the interevent interval of spontaneous currents from **(A, B)**, respectively **(F, H)** Normalized currents from **(A, B)**, respectively.

We also tested the effect of ChC on the hypoxia-induced increase of spontaneous NMDA retinocollicular activity. As with the evoked neurotransmission, we added ChC during the reoxygenation (*n* = 8), and during hypoxia (*n* = 7). In the first case, we observed the hypoxia-induced decrease of the currents decay time constants in the background of the increase in currents amplitudes. The presence of ChC during reoxygenation restored both parameters to the basic level (Figures 3A, C, F; decay time constants during control 43.7 ± 8.2 ms; hypoxia 15.6 ± 3.8 ms; reoxygenation in presence of ChC 42.8 ± 6.5 ms; *t* = 18.5, *df* = 60, *p* < 0.0001 – hypoxia compare to control; *t* = 0.4, *df* = 38, *p* = 0.7 – reoxygenation compare to control). In the second case, ChC abolished the hypoxia-induced changes of spontaneous currents amplitudes and decay time constants (Figures 3B, D, H; control 37.3 ± 4.9 ms; hypoxia in the presence of ChC 39.3 ± 5.8 ms; reoxygenation 38.5 ± 6.1 ms; *t* = 1.1, *df* = 50, *p* = 0.2 – hypoxia compare to control; *t* = 0.6, *df* = 33, *p* = 0.6 – reoxygenation compare to control). In both cases, the hypoxia-induced increase of the occurrence frequency of spontaneous events remains elevated despite the presence of ChC. The quantitative analysis of changes in the frequencies of spontaneous NMDA currents is depicted on the cumulative probability plots (Figures 3E, G).

We have also pointed out that the presence of ChC under normoxic conditions didn't induce any changes in spontaneous current amplitudes, their kinetic characteristics, distribution, or the occurrence frequency of spontaneous events.

## 4. Discussion

Our results show that two phenomena underlie hypoxia-induced LTP of NMDA retinocollicular neurotransmission: (1) the shortening of PSCs kinetics and (2) the involvement of the PKC signaling pathway in its induction and maintaining.

Functional NMDARs are heteromeric assemblies of NR1, NR2, and NR3 subunits. Each subunit composition of the receptor demonstrates distinct functional and electrophysiological properties (Cull-Candy and Leszkiewicz, 2004). The NR2 subunits determine such functional properties as the open probability of the receptor, its high affinity for glutamate, modulation by glycine, sensitivity to voltage-dependent block by Mg^2+^, and current kinetics (Perin-Dureau et al., 2002; Cull-Candy and Leszkiewicz, 2004; Hatton and Paoletti, 2005; Paoletti and Neyton, 2007). The changes in NR2 subunits composition lead to changes in electrophysiological characteristics of NMDA currents and vice versa (Vicini et al., 1998; Roberts and Ramoa, 1999).

The shortening of NMDA currents in the background of hypoxia-induced elevation of amplitudes we observed might reflect changes in NMDAR subunit composition. Using decay kinetic characteristics and literature-based analysis, we considered that the decrease of the PSCs decay time constant might be caused by an increase in the NR2A/2B ratio (Vicini et al., 1998; Philpot et al., 2001; Xue et al., 2010). Because of stronger glutamate binding affinity and longer duration of currents, NR2B containing NMDARs are more permeable for calcium than NR2A containing ones (Flint et al., 1997; Vicini et al., 1998). Therefore, the increase in the NR2A/2B ratio should decrease calcium influx during hypoxia-induced potentiation of NMDA retinocollicular neurotransmission. Moreover, using some statistical models, authors have declared that NR2A may not only shorten the decay time but also increase current amplitudes (Iacobucci and Popescu, 2017). In our research, there was no statistically significant synchronization between the increase in current amplitudes and the decrease in the decay time. Due to the short period of hypoxia duration, subunits changes might be associated with lateral receptor mobilization from adjacent locations, rather than with a new subunits expression (Baez et al., 2018). Overall, we tend to consider the shortening of the current kinetics as a compensatory mechanism in the background of pathologically-induced long-term plasticity as it aims to decrease calcium influx.

We also observed that the presence of ChC completely blocked hypoxia-induced LTP of NMDA retinocollicular neurotransmission and the elevation of the amplitudes of spontaneous NMDA events. ChC is the most recognized potent and specific inhibitor of PKC for isoforms α and β (Herbert et al., 1990; Chmura et al., 2000). The activity of PKC in hypoxic injury has been shown in different tissues. But the distinct role of PKC in cell response to hypoxia as well as the type of isoforms involved in it are still controversial (Skaper et al., 2001; Matsumoto et al., 2004; Bright and Mochly-Rosen, 2005). We showed that the blockade of PKC activity is able not only to prevent pathological hypoxia-induced LTP of NMDA neurotransmission but also to reverse synaptic strength from the potentiated to basal, pre-LTP level. The reversal of LTP is called depotentiation. In contrast to activity-dependent synaptic plasticity that underlies such higher cognitive processes as learning and memory, depotentiation may be responsible for forgetting or mediating degenerative disorders (Huang et al., 2001; Babür et al., 2018). Besides, depotentiation might be involved in developmental changes of synaptic transmission (Huang et al., 2001; Qi et al., 2013; Tao et al., 2019). However, there is no evidence of what role it may play in pathologically-induced LTP.

We also found that ChC blocked the initiation of hypoxia-induced shortening of NMDA evoked and spontaneous currents. It is interesting that, already induced shortening of evoked NMDA PSCs showed irreversibility despite the presence of ChC, whereas spontaneous currents showed the ability to reverse their kinetic to the initial duration. PKC activity is thought to play a key role not only in such functional changes of NMDAR as an increase in the amplitude of currents, channel open probability, and current kinetics but also in trafficking and inserting the receptors into synaptic membranes (Gerber et al., 1989; Chen and Huang, 1992; Xiong et al., 1998; Lan et al., 2001). The existence of such a difference in the reversibility of evoked and spontaneous current kinetics proves the differential regulation of synaptic and extra-synaptic NMDAR (Li et al., 2002; Hardingham and Bading, 2010). The blockade of the PKC signaling pathway didn't affect the hypoxia-induced increase in the occurrence frequency of spontaneous NMDA events.

In this study, we demonstrated that the PKC signaling pathway mediates hypoxia-induced LTP of NMDA retinocollicular neurotransmission at the expression and maintenance stages. According to the literature, PKC might affect NMDAR function directly by phosphorylation of its subunits (Lan et al., 2001; Liao et al., 2001; Zhou et al., 2021) or indirectly by interacting with molecules related to LTP including calcium/calmodulin-dependent protein kinase II (CaMKII) (Gardoni et al., 2001; Yan et al., 2011), postsynaptic density protein (PSD-95) (Wang and Peng, 2016), etc. Moreover, the PKC might act pre- or postsynaptically (Soderling and Derkach, 2000; Brager et al., 2003). Identification of precise mechanisms of PKC-mediated LTP of NMDA retinocollicular neurotransmission will reveal molecules, or complexes involved in hypoxia injury.

The physiological significance of our research can be summarized in the following, (1) hypoxia-induced LTP of NMDA retinocollicular synaptic transmission is associated with the shortening of PSCs, which is a potential cellular compensatory mechanism (2) The PKC signaling pathway mediates both hypoxia-induced LTP and associated changes in current kinetics (3) Moreover, the blockade of PKC by ChC leads to depotentiation of hypoxia-induced LTP. We consider that under pathological conditions, such depotentiation reflects the ability of the system to restore normal functioning, and ChC is a promising agent for the prevention and treatment of hypoxia-induced lesions of the retinocollicular neurotransmission (4) Our results provide the necessary electrophysiological platform for further research.

## Data availability statement

The raw data supporting the conclusions of this article will be made available by the authors, without undue reservation.

## Ethics statement

The animal study was reviewed and approved by the Ethics Committee of Bogomoletz Institute of Physiology National Academy of Science of Ukraine.

## Author contributions

HD and NV contributed to the conception and design of the study. HD performed the experiments, analyzed the data, and prepared the manuscript. All authors read and approved the final manuscript.
